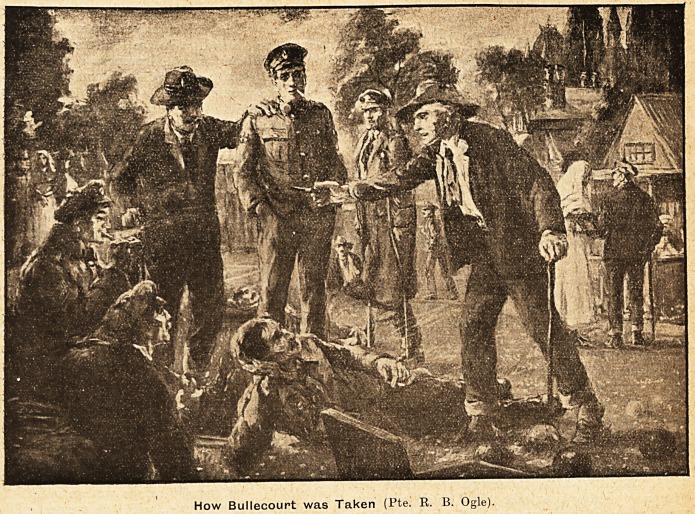# Village Practice.—II

**Published:** 1917-12-22

**Authors:** 


How Bullecourt was Taken (Pte. R. B. Ogle).

### VILLAGE PRACTICE.—II.

### An Autobiographical Fragment.

WITH a certain amount of surprise I received an intimation that the first instalment of these notes would be printed, and also a request that I would send more. I can only say I am prepared to go on as long as the Editor and readers will stand it! Native caution prevented me giving anything in the nature of an outline of the course I intend to pursue in these sketches. It would have been humiliating to find at the end of my preface of intended procedure the fatal blue pencil making some more or less caustic remark, such as "Oh! no, you don't"! Since it appears probable, however, that provided my wits hang together you and I, gentle reader, may renew our acquaintance from time to time, it may be advantageous that I should state briefly the intended nature and scope of the fragments. There will, then, be unfolded no ordered march of events. I propose to follow no fixed scheme or plan, wishing rather to take you with me on an autobiographical ramble. Probably the slightest experience through which you and I pass has its lesson, and is allotted a place in the general scheme of things. And we shall be wise if, each in his own life, we scrutinise with care the most ordinary and apparently insignificant stages of our journey down the long road. For the great crises of life do not by any means always appear with an arresting fanfare of trumpets and gorgeous State ceremony. Oftentimes a most critical event, upon our right or wrong treatment of which the whole future of a career may depend for its making or marring, appears before us clothed in very drab apparel. How often has one heard a man or woman say, "Ah! years ago my chance came, but I did not seize it. I can see now that my whole future depended upon the step I took then, but at the time I did not realise the importance of the decision. It seemed to be such a trivial thing, and I acted without sufficient thought and deliberation, with the result that I have regretted it to this day"! Yes, there is not always a high wall to obstruct our progress and to turn us aside. There may be but the faintest indication of another path. The voice is not always in the whirlwind, and the soul must be attuned to the music of the spheres if one would not miss the whispered "This is the way, walk ye in it."

"NO RAILS."

But whilst this is so, such points are rather of individual than general importance, and I do not propose to enter in these sketches into minute points of this kind, although our ramble is to be an autobiographical one. Rather shall I endeavour to pick out from the mass

☛ The first article appeared on November 24.

VILLAGE PRACTICE—(*continued*).

of detail experiences that are likely to be of general interest and adaptation. And we will be the slave of no almanac, but will wander up and down the highway of the past three years fancy free, now discussing together an experience of, say, 1916, and anon gossiping in 1915 or 1917. Inclination and the impulse of the moment shall alone be our guide. And I shall endeavour to realise the companionship of my readers, talking not *at* you, nor even entirely *to* you, but, so far as circumstances of the moment allow, *with* you. When I hear one of you say quietly to another "He's rambling with a vengeance; here we are clean off the main track, goodness knows where," then I shall remind you that we started out with the stipulation "No rails"!

### A Revolution for the Better.

It is inevitable that, in any survey of the past three years' work and experiences, we shall find our meditations coloured by events far afield. The very atmosphere is electrically charged. Men and women in country as well as in towns have been stirred into alertness, watchfulness, and responsiveness to the thrilling happenings of the time as never before. Thought and action have become keen, swift, and yet wary, for lethargy has been shaken and wildness restrained by the spirit and demands of the hour. And this quickening and controlling have influenced the whole life. The years as they pass take toll of us all, and even three years of ordinary quiet life in peace-time bring a certain change and development in some one or more directions. But the change in the people amongst whom I have worked during this time is to be regarded as due not so much to evolution as to revolution. And the change is, speaking generally, for the better. There is a pensiveness and also a doggedness, a sweetness and also a strength, and withal a simplicity discernible in many of those of mature years which was absent three years ago. We are all touching life and experience at vital points to-day, and are living very near to the heart of things. First principles have emerged which for long had lain neglected, cloaked with the miserable rags of convention. The tempest has stripped every shred of unworthy covering from the underlying eternal realities. Our cosmos has been blasted into chaos, and truth, naked and rugged, rises from the wreck and makes its appeal with an importunity that will not be denied. Problems and questions have to be faced and some answer found. Men and women want to know things about which they were, perhaps, previously indifferent.

### Does Everybody Think?

Before the war a great many people put out their thinking as they do their washing, getting others to do it for them. This is not so to-day; we are beginning to think for ourselves. The drama of life is appealing with insistency to us all. The first impact found us unprepared and we were thrown back in disorder, but we have braced ourselves for issues we know cannot be shirked. We are "girding up the loins of our mind"; mental looseness and dilatoriness are giving way to purposefulness and concentration, and, as a people, I believe that we are awaking to the magnitude of the problems—personal, social, political, religious—that clamour for recognition. In many the awaking from sleep is a long and tedious process, and in a great many cases a groping drowsiness may be all that can be expected. But the holy quest is there.

### A Woman of Ninety Years.

This very afternoon I visited an old village woman who is in her ninetieth year, and the following dialogue took place: "Well, Grannie, how are you getting on?"—"I feel better, thank you, sir." "Did you have a good night?"—"No, I didn't sleep much." "What kept you awake, pain?"—"Oh! no, I didn't have any pain much." "What was it then?"—"Well, I think it is so much studying." The quaintness of the expression from one in her circumstances brought a smile to the lips, and, blind to its significance, I said, "I suppose you think about bygone days when you were younger." Like a flash came the reply, "Oh! no, I don't think about them things; I think about all of them boys fighting out there, and I wonder about it all. I wonder about it all!" And the old village woman is in fellowship, heart communion, with the greatest philosopher—"I wonder about it all!"

### The Birth of Soul Consciousness.

Yes, this and no other age is the age of the renascence of wonder. Does any father ever forget the first time he saw "wonder" looking out through the eyes of his little child? It is the birth of soul consciousness, and, in a deeper sense, to-day we are becoming as little children, we are beginning to wonder. Thus the deep shadow of the world's tragedy is over all, influencing and colouring all thought. Outwardly in these remote places things are the same as of old. Intrinsically they are different. Here, as I write, all is peaceful on the surface, and no sound is to be heard save the scratching of my pen. Out yonder the earth is rocking with the shock of battle. And as I close this fragment there come to my mind those words from the "Elegy":

> Now fades the glimmering landscape on the sight,
> And all the air a solemn stillness holds.

To-day the music of those words is instinct with the note of destiny. Old things are fading, passing, and the night is upon us; but, with hopefulness and confidence, though also with strained eagerness and bated breath, we, who for one reason or another have been kept back from the blood and sweat of the struggle, are awaiting, in the solemn stillness, the birth of a New Day.

(*To be continued.*)

## Figures and Tables

**Figure f1:**